# Angular flux creep contributions in YBa_2_Cu_3_O_7−δ_ nanocomposites from electrical transport measurements

**DOI:** 10.1038/s41598-018-24392-1

**Published:** 2018-04-12

**Authors:** F. Vallès, A. Palau, V. Rouco, B. Mundet, X. Obradors, T. Puig

**Affiliations:** Institut de Ciència de Materials de Barcelona, ICMAB-CSIC, Campus UAB, 08193 Bellaterra, Spain

## Abstract

The shape of the electric-field—current-density (E-J) curve is determined by flux pinning and also by dynamics of vortices. Here, we propose a novel methodology to study the normalized flux creep rate S in YBa_2_Cu_3_O_7−δ_ measured from E-J curves obtained by electrical transport measurements that provides a fast and versatile way to foresee the flux magnetic relaxation in films and disentangle angular flux creep contributions by the scaling of the isotropic contribution of S. After a detailed comparison of various pristine and nanocomposite films with differentiated nanostructures, we focus on the roles that intrinsic pinning and stacking faults (YBa_2_Cu_4_O_8_-intergrowths) play when the magnetic field is applied parallel to the superconducting CuO_2_ planes. This study reveals that the emerging intergrowths provide advanced pinning properties that additionally reduce the thermal activated flux magnetic relaxation. For this purpose, creep analysis becomes a very appropriate tool to elucidate the dominance of the different pinning sites at different regions of the magnetic-field—temperature diagram.

## Introduction

The introduction of controlled nanoscale defects within YBa_2_Cu_3_O_7−δ_ (YBCO) has been demonstrated to improve the flux pinning properties and enhance the critical current density J_c_ at high magnetic fields. However, it has been shown in high temperature superconductors (HTS) that time and temperature evolution of the critical current are both governed by the pinning and dynamics of vortices, revealing that the magnetic phase diagram is a result of different competing energies, pinning and thermal energies among them^1,2^. Thermally activated flux creep causes logarithmic time decay on the critical current and influences the voltage-current characteristics, especially at higher temperatures in detriment of possible applications for HTS. A deep study on this topic is not only necessary from a fundamental point of view, but also to acquire technological knowledge for the electrical performance of second generation coated conductors^3–5^. In particular, the decay of critical current with time in persistent mode applications and flux jumping in high-field magnets have become very significant issues. In recent years, several studies in nanostructured HTS have been concentrating on the interplay between defects and vortex dynamics, mainly by the use of magnetic relaxation inductive methods when the magnetic field is applied parallel to the crystallographic c-axis^2,6–9^. However, a more powerful tool is needed to elaborate these studies in a faster way which moreover enables to expand this analysis to other orientations of the magnetic field.

The standard flux creep model^10^ together with the logarithmic current dependence of the pinning potential proposed by Zeldov *et al*.^11^ leads to an approximation where the electric-field—current-density E-J dependence can be described by a power-law relation E ∝ J^N^ in the region of flux creep, where the index N is proportional to the pinning potential and inversely proportional to the thermal energy: N ∝ U_P_/k_B_T. Yamasaki and Mawatari^12^ went further with the study of the E-J characteristics and implementing Faraday’s law for N ≫ 1, postulated that the normalized flux creep rate S can be related to the index N as S = −∂ ln|J_c_|/∂ ln(t) = −∂ ln|M|/∂ ln(t) = 1/(N−1), where M is magnetization. Hence, in this approximation, S is nearly inversely proportional to N. Therefore, studying E-J characteristics measured by electrical transport measurements we are able to measure flux creep values coming from the slope of the logarithmic current-dissipation dependence. However, we need to consider that creep rates from electrical transport measurements slightly differ from rates calculated with inductive measurements, although they portray similar features and preserve the same order of magnitude (see Supplementary Fig. S1). The reason of this disparity is given by the use of a different criteria: the time scale of the measurement is shorter in transport measurements together with the fact that the generated electric field is higher^13^. Furthermore, the dynamics of vortices will vary from applying a constant field with a corresponding induced current that relaxes with time (in inductive measurements) than applying an increasing current as a driving force (in transport measurements). Therefore, results in both methods should not be exchanged. The powerfulness of this approach lies in the direct, very fast and versatile way to sense the essentials of magnetic relaxation from the derivative of the current-voltage characteristics and the capacity to broaden this study to other field orientations, unreachable by other methods.

## Results and Discussion

**Figure 1:**
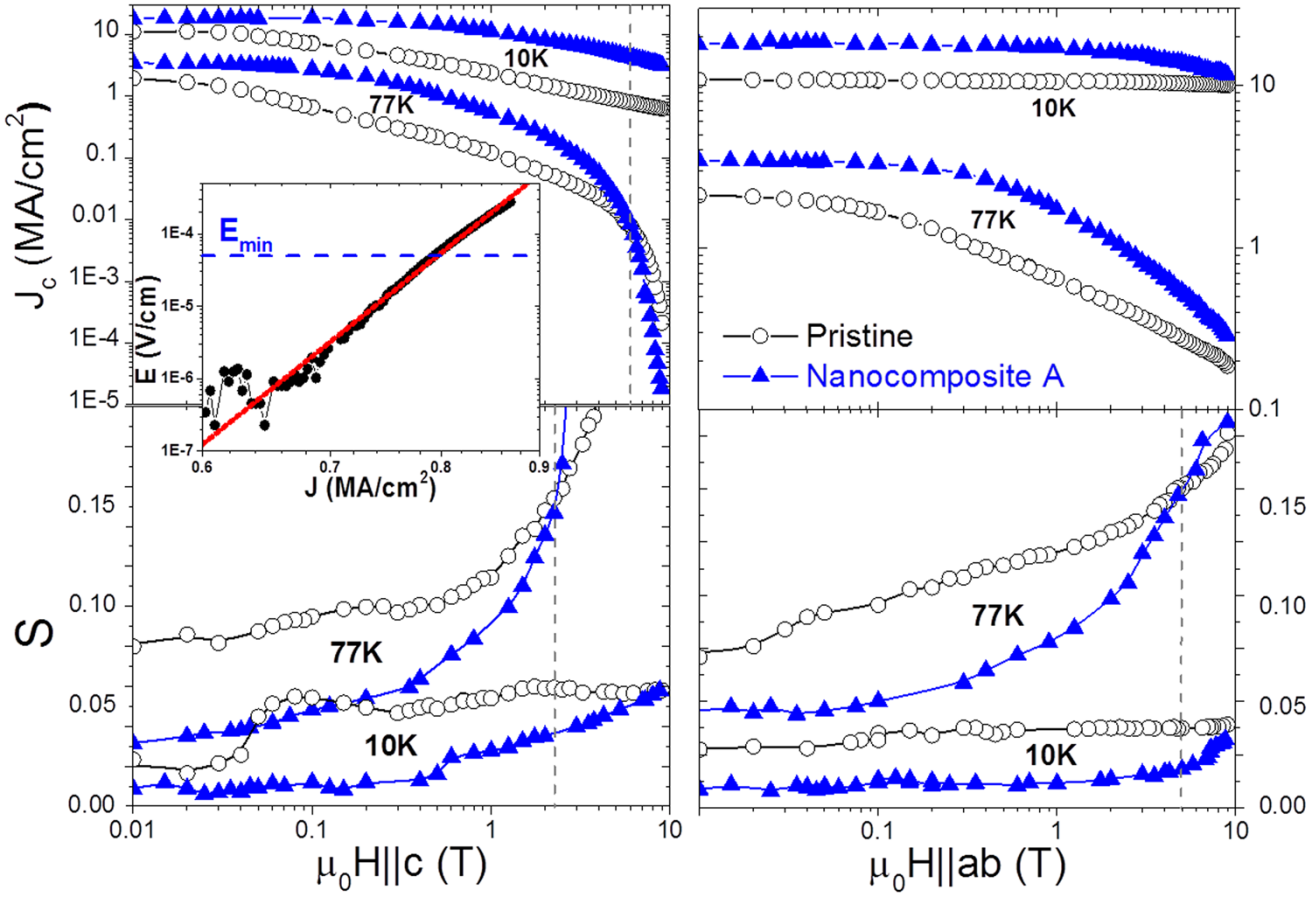
Field dependence of Jc and S for H||c and H||ab. The curves were measured for a pristine (open) and nanocomposite A (closed) at 77 K and 10 K. Dashed lines indicate the field where the 77 K curves intersect. The inset shows a particular estimation of N from the E-J dependence at 10 K and 7 T (||c) for the pristine sample.

In this study, we present results obtained by electrical transport measurements on YBCO thin films grown by chemical solution deposition with and without the inclusion of nanoparticles in the YBCO matrix, whose growth details were reported previously^14^. We have studied nanocomposites from three different groups (I, II and III) as will be explained below, regarding their disparate concentrations of Stacking Faults (SFs) caused by distinct nanoparticle compositions and processing methods. We focus on the results obtained in nanocomposites named A, B, C, and D in comparison to pristine films, where nanocomposites A, B and D have a great number of SFs whereas nanocomposite C has a rather low amount, observed by scanning transmission electron microscopy (STEM) (see Supplementary Fig. S2). The N value was calculated by fitting the power-law relation E ∝ J^N^ over a range extending from a minimum electric field E_min_ of 50 μV/cm to a maximum electric field of ~300 μV/cm where the measurement stops (see inset in Fig. 1), ensuring that we measure N close to J_c_ in the region of flux creep and that we have enough data for a proper fit (see Supplementary Fig. S3).

Figure 1 depicts J_c_(B) and S(B) dependences for the two main orientations (0° and 90°) for representative pristine and nanocomposite samples. The normalized relaxation rate was calculated from the equation S = 1/(N − 1). At 77 K, the nanocomposite shows the expected enhanced J_c_ and reduced S at low and intermediate fields in comparison with the pristine sample. However, an intersection at high magnetic fields takes place. Whereas the intersection for J_c_ occurs at 6 T (||c) or at ~30 T (||ab) after extrapolation, for S it happens at an earlier stage, precisely at 2 T (||c) and 5 T (||ab). The intersection appears when approaching the irreversibility line where vortex melting turns up. We attribute these results to the fact that some nanocomposites are more affected at this field-temperature regime by the coherence breaking of correlated defects (twin boundaries)^15,16^, which is earlier sensed with flux creep measurements. At 10 K, the intersection is shifted to higher magnetic fields, enlarging the region where the nanocomposite provides simultaneously higher pinning and lower creep.

**Figure 2:**
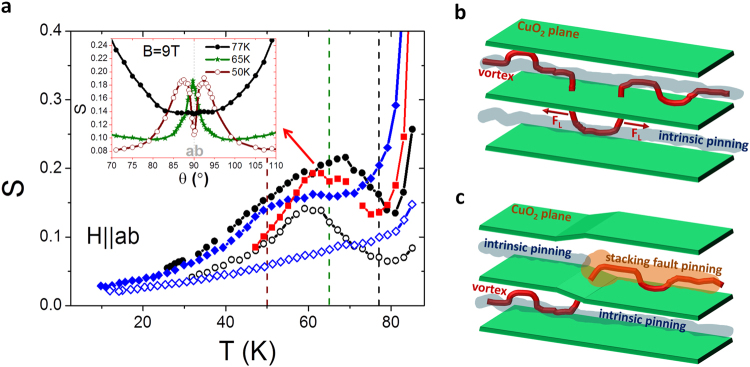
Temperature dependence of S and vortex representations. On the left, (**a**) temperature dependence of S for H||ab at 1 T (open) and 9 T (closed) for a pristine sample (circles) and nanocomposites B (diamonds) and C (squares). The inset shows the S(θ) dependence at different temperatures (dashed lines in the figure) for nanocomposite C. On the right, representation of (**b**) the formation of a double kink given by periodical and regular IP and (**c**) the formation of a kink in the modified CuO_2_ planes scenario caused by the emergence of a SF. F_L_ is the Lorentz-like Force driven by the interaction between the flux line and the applied current.

Let us consider next the temperature dependence of the flux creep rate S when the magnetic field is applied parallel to the ab planes, motivated by the purpose of studying the interplay of the two main pinning centers acting in this orientation: intrinsic pinning (IP) and SF pinning. Fig. 2(a) shows S(T) for a pristine sample and two nanocomposites with different concentrations of SFs at μ_0_H||ab = 1 T and 9 T. Comparably to the effect described with the field dependence in Fig. 1, the values of creep relaxation are decreased in nanocomposites except when approaching the irreversibility line, i.e. at very high temperatures. As regards the temperature dependence, besides the monotonic increase of S with increasing temperature, we observe an S(T) peak appearing in the region that comprises 50 K to 80 K, especially notable for the pristine sample and nanocomposite C. For nanocomposite B, the one with more SFs, the peak reduces or even vanishes at low magnetic field, confirming that the presence of artificially induced SFs modifies the structure of the CuO_2_ planes and its effect on thermal activation, obtaining lower creep rates for a large range of temperatures. Similar S(T)-peaks (also called S(T)-dips in other works, since both shapes occur concurrently with each other) have been observed in YBCO single crystals and thin films, attained by inductive measurements for H||c, where the peak has been attributed to the generation and expansion at no energy cost of vortices, mediated by double-kink (DK) thermal excitations in correlated pinning sites as columnar defects^6,17^, twin boundaries^8,18^, or by a similar effect described in nanoparticles, where the vortex line slides along the surface of the nanoparticle^9^. We propose by the following explanation, that DKs also account for the S(T)-peak arising for H||ab, aided by the periodical and regular IP originated between the superconducting CuO_2_ planes (depicted in Fig. 2(b)). Dissipation due to DK excitations in the regime of IP can be detected by performing J_c_(θ) electrical transport measurements around H||ab and analyzing the obtained I-V curves^19–25^. In these experiments, an inverse correlation between J_c_ and N was obtained, with a J_c_(θ) dependence showing a peak for H||ab whereas N(θ) is showing a dip. This inverse correlation is attributed to a loss in the pinning energy, U_p_, due to the formation of DK excitations when vortices lie parallel to the ab planes. The relation between the N(θ)-dip (implying an S(θ)-peak) and the S(T)-peak is shown for nanocomposite C in the inset of Fig. 2(a). At 77 K we find, as expected for a direct J_c_-N correlation, a minimum in the S(θ) curve at θ = 90°. Decreasing temperature, we approach the temperature T^⋆^ where the vortex core diameter 2ξ_c_ drops below the periodic separation of CuO_2_ planes, that for the YBCO triple perovskite follows the equation:

$$2\frac{{{\boldsymbol{\xi }}}_{0}}{\sqrt{1-{{\boldsymbol{T}}}^{\star }/{{\boldsymbol{T}}}_{c}}}=\frac{2}{3}{\boldsymbol{d}}$$

where d corresponds to 1.168 nm^26^. In our samples we obtain T^⋆^ ~ 60 K. Thus, close to T^⋆^, at 65 K vortices start to be pinned by the intrinsic single pinning wells so that DK excitations take place from one pinning center to the other. Therefore, we observe the appearance of the S(θ)-peak at this temperature, which spans an orientation width of 5° and widens as temperature decreases. However, at low temperatures (50 K in this case), a narrow S(θ)-dip arises at the center of the S(θ)-peak, indicative of a lock-in transition^27^, also reported in other electrical transport studies^23,25^. This latter effect shows that at low temperatures, the thermal energy k_B_T is not high enough to cause movement of vortices through DK formation when they lie almost parallel to the CuO_2_ planes. On the contrary, vortices get locked between the planes.

We have seen that for the observation of the S(θ)-peak when θ = 90°, temperature and magnetic field are key conditions. However, the appearance of the S(θ)-peak does not occur at the same conditions for films with different microstructures, meaning that the interplay between the CuO_2_ planes (origin of IP) and the generated planar SFs is critical for the observation of these thermal excitations.

**Figure 3:**
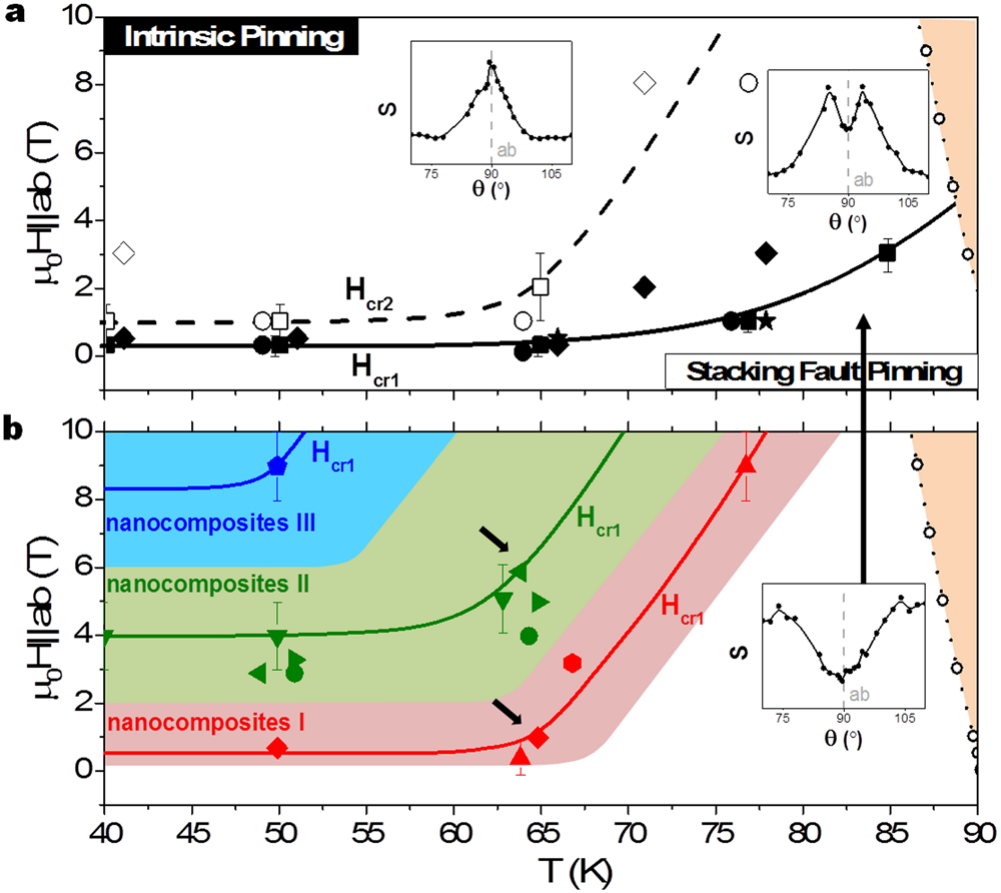
Magnetic-Temperature phase diagram for H||ab. The closed symbols (experimental results) and solid lines (guides to the eye) represent the lower crossover line μ_0_H_cr1_(T) for (**a**) pristine and (**b**) nanocomposite films. The open symbols (experimental results) and dashed line (guide to the eye) represent the upper crossover line μ_0_H_cr2_(T). The dotted line with open circles refers to the experimental irreversibility line. Crossover points are plotted for a large variety of samples, identifying three typologies of nanocomposites (in three different colors) regarding the region of μ_0_H_cr1_(T). The insets show the S(θ) dependence for a pristine sample in the regions of the diagram separated by the crossover lines in figure (**a**).

By analyzing the S(θ, H, T) dependencies, we have determined a lower crossover line μ_0_H_cr1_(T) in the H-T diagram separating creep regions for a variety of pristine samples (see Fig. 3(a)). In the first region, at high temperatures and low magnetic fields, we observe an S(θ)-dip. In the other limit, at low temperatures and high magnetic fields, we find an S(θ)-peak. The transition from one region to the other is in general not abrupt, defining a region in-between where the peak is not completely formed below the upper crossover line μ_0_H_cr2_ (see insets of Fig. 3(a)). As commented before, the presence of the S(θ)-peak is related to the thermal excitations aided by IP. Thereby, for H||ab the region above the lower crossover line is dominated by IP, whereas the region below is dominated by pinning caused by defects that destabilize the regularity and periodicity of IP thus avoiding DK formation. In our films, we have observed that SF pinning consisting of YBa_2_Cu_4_O_8_(Y248)-intergrowths is the main responsible mechanism of such disruption. In Fig. 3(b), we depict the lower crossover line μ_0_H_cr1_(T) found for nanocomposite films, whose density of Y248-intergrowhts is much larger than that one of pristine films, as confirmed by STEM observations^28^ and also indicated by the broadening of the Jc(θ)-peak for H||ab^29^. The crossover happens to be shifted to higher fields and lower temperatures in the diagram. This shift is consequently given by the changes in the nanostructure. In fact, a structure dominated by the nanoparticles-emerging SFs strongly modifies the periodic scenario of the CuO_2_ planes responsible for IP by means of distortion of the planes and generation of a stronger pinning region that avoids DK excitations (see Fig. 2(c)). The general trend that we observe studying a large variety of nanocomposites is that SF pinning dominates in a wider range of the diagram whenever we introduce nanoparticles, leading to small distortions in several films (nanocomposites I), to bigger distortions for the majority of the studied films (nanocomposites II), and dominating the whole studied diagram in some particular cases (nanocomposites III).

**Figure 4:**
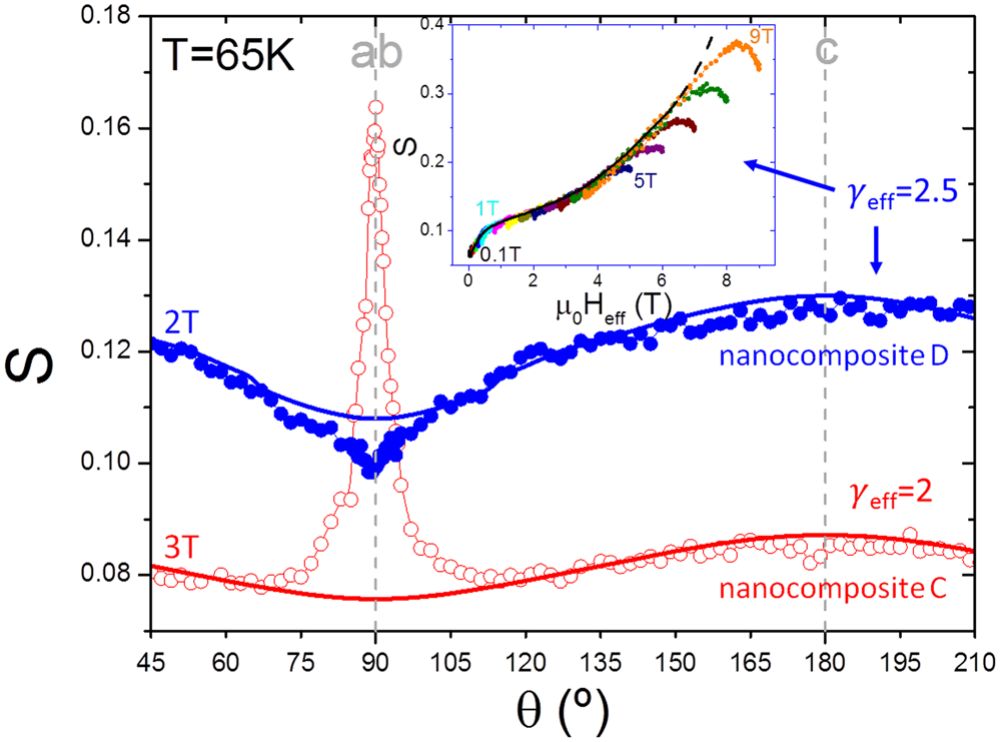
Angular dependence of S and Siso. The curves were measured at 65 K at 2 T and 3 T for nanocomposite C (open) and nanocomposite D (closed), belonging to nanocomposites I and nanocomposites II in Fig. 3, respectively. The solid lines represent the isotropic contribution S^iso^(θ), obtained from the scaling S^iso^(H_eff_(θ)) as shown in the inset. The inset shows S(θ) as a function of H_eff_ for nanocomposite D at 65 K from 0.1 T until 9 T with γ_eff_ = 2.5. The black solid line corresponds to the collapse of S^iso^ (H_eff_).

Similarly to the methodologies presented for J_c_ in other studies^30,31^, we confirm that the Blatter scaling approach^32^ can be also applied for S, strongly widening the possibilities used until now with inductive measurements to evaluate flux creep mechanisms, especially in YBCO films and nanocomposites where different pinning centers coexist with distinct contributions that may be separated by angular dependent results. The inset of Fig. 4 shows the isotropic collapse of S when plotted as a function of the effective magnetic field H_eff_ = H(cos^2^θ + γ_eff_^−2^sin^2^θ)^1/2^ at 65 K for a nanocomposite sample with an effective anisotropy γ_eff_ of 2.5. This low γ_eff_ is comparable to the value obtained from J_c_ measurements, where we demonstrated that it is caused by the nanoscale strained regions generated at the surroundings of the 248-intergrowths^28^.

For the collapse of S, it is important to remark though, that thermal excitations of different nature (for example, those generated in twin boundaries) may provoke large changes in S, extending in some cases (in particular, for some pristine samples at 40–65 K) to a very wide range of orientations, causing the inability to scale the isotropic contribution. Nanocomposites mostly inhibit the formation of large thermal excitations and hence the collapse of the isotropic contribution of flux creep S^iso^ is in general clearly observable. For comparison, we have applied this methodology into two different nanocomposites that are pointed out with small arrows in the diagram of Fig. 3(b). The angular dependences of S and S^iso^ of these samples are plotted in Fig. 4. We observe that the appearance of the S(θ)-peak for H||ab, attributed to DKs jumping across the regular intrinsic pinning centers, corresponds to the anisotropic flux creep contribution S^aniso^. Being both samples measured at similar conditions, we observe for this orientation that the nanocomposite D is dominated by SFs whereas the nanocomposite C is dominated by IP, showing a decrease and an increase given by S^aniso^, respectively. The decrease indicates the effect of correlated pinning centers hampering the vortex flux motion whereas the increase indicates favoring of thermal flux motion aided by the regular and recurring pinning centers. Notice that the effect in the nanostructure given by the insertion of nanoparticles is different depending on the nanoparticle composition and growth conditions commented in other works^14,28^. In nanocomposite D, a higher amount of SFs emerge from the incoherent interface between nanoparticles and YBCO, causing a stronger distortion of the CuO_2_ planes and adding new pinning sites that turn out to be the dominant (see Supplementary Fig. S2). On the other hand, for H||c we do not observe either an increase or a decrease for both nanocomposites, suggesting that the generation of SFs breaks the twin boundary coherence and avoids flux jumping across these correlated defects^33^.

**Figure 5:**
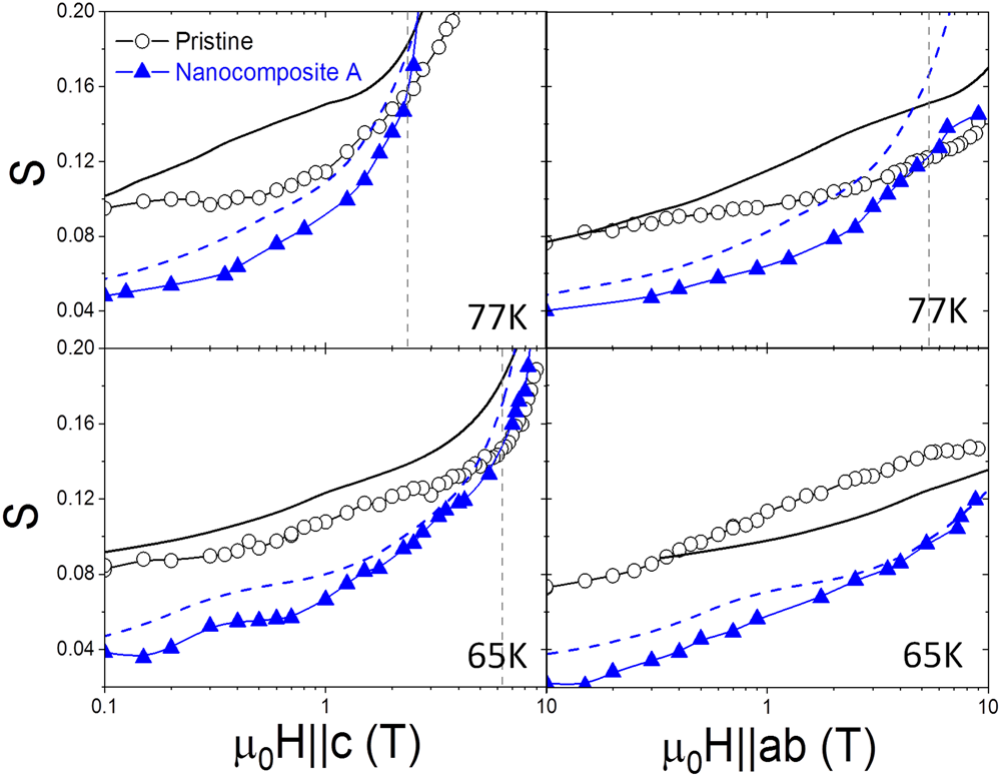
Field dependence of S and Siso. S (experimental points) and S^iso^ (simple curves) are plotted for H||c and H||ab for a pristine (open, solid) and a nanocomposite (closed, dashed) at 77 K and 65 K. Straight dashed lines show the magnetic field where the S curves intersect.

In order to further analyze the different contributions to the relaxation mechanisms, Fig. 5 shows S and S^iso^ versus magnetic field at 77 K and 65 K for the pristine and nanocomposite samples shown in Fig. 1, being nanocomposite A one of those from nanocomposites II. For H||c, S^iso^ exceeds the absolute S continuously, specially for the pristine film, indicative of a decrease of relaxation induced by correlated defects acting as pinning centers and preventing flux relaxation, in agreement with the observation of twin boundaries in this type of films^15^. For H||ab we observe a similar situation for the nanocomposite, where SF pinning dominates, thus preventing flux relaxation. However, the pristine undergoes a drastic change from 77 K to 65 K. DK excitations start being formed at lower temperature when the vortex core size fits the intrinsic pinning spacing and boost thermal flux activation causing an increase of flux creep. We also observe that all intersections in terms of S and S^iso^ for H||c and H||ab between the pristine and the nanocomposite are shifted to higher magnetic fields when reducing temperature and moving away from the irreversibility line. Moreover, the capability of the nanocomposite of preserving a decrease of S coming from S^aniso^, results in S values that are far below the ones that we obtain in the pristine sample at the temperature of 65 K.

## Conclusions

In summary, we have investigated for first time the magnetic flux relaxation mechanisms of S(θ, H, T) from electrical transport measurements in YBCO thin films grown by chemical solution deposition with special focus on the comparison between pristine and nanocomposite samples with the inclusion of nanoparticles and SFs. The unveiled capability to scale the isotropic contribution of flux creep is of prime importance for understanding the correlation between flux dynamics and microstructure. We have implemented this methodology to study the role of correlated pinning parallel to the ab-planes, realizing that SFs not only provide additional pinning but also weaken thermal flux activation, offering the chance to avoid flux creep effects. We have seen that the separation between S^iso^ and S^aniso^ also permits the identification of thermal excitations originated by correlated pinning. We suggest that the implementation of this analysis to a broader typology of layered superconductors can shed new light on the interplay between microstructure and vortex matter and be crucial for the technological implementation of superconducting films in the use of coated conductors.

## Methods

Epitaxial c-axis oriented YBCO thin films were grown by chemical solution deposition from metal organic precursor solutions following the trifluoracetate (TFA) route on single crystal LaAlO_3_ substrates. Nanocomposites have been achieved by adding extra metal salts to the YBCO precursor solution to grow nanoparticles via spontaneous segregation during the YBCO growth. The secondary phases used for this study were Ba_2_YTaO_6_ and mixed compositions of Ba_2_YTaO_6_-CuO, Ba_2_YTaO_6_-Y_2_O_3_, Ba_2_YTaO_6_-BaZrO_3_ and BaZrO_3_-Y_2_O_3_ with different molar concentrations ranging from 6% to 15%, leading to disparate pinning landscapes with densities of SFs in the range of 3·10^−4^–4·10^−3^ nm^−2^, observed by STEM.

The thickness of the superconducting films varies from 200 to 300 nm, analyzed with a Profilometer P16+ from KLA Tencor. The current-electric-field characteristics were evaluated in a Quantum Design PPMS system provided with a 9 T magnet with temperature variable down to 4.2 K. Silver contacts were deposited in a sputtering system from TSST and subsequently post-annealed, ensuring resistances below 10 μΩ·cm^2^. The films were trimmed into 10–50 μm narrow bridges with lengths of 200 μm by standard optical lithography with a Micro-Writer from Durham Magneto Optics LTD and wet etching in H_3_PO_4_.

Electrical transport measurements were carried out by the standard four-point method. The current was applied parallel to the ab-plane, always perpendicularly to the magnetic field which was rotated with angle θ from the c-axis (0°) to the ab-plane (90°), ensuring maximum Lorentz force configuration. J_c_ was determined for a 10 μV/cm electric field criterion.

### Data availability

The data that support the findings of this study is available from the corresponding author upon request.

## Electronic supplementary material

Supplementary Information
