# Author Correction: Angular flux creep contributions in YBa_2_Cu_3_O_7−δ_ nanocomposites from electrical transport measurements

**DOI:** 10.1038/s41598-018-25540-3

**Published:** 2018-05-01

**Authors:** F. Vallès, A. Palau, V. Rouco, B. Mundet, X. Obradors, T. Puig

**Affiliations:** Institut de Ciència de Materials de Barcelona, ICMAB-CSIC, Campus UAB, 08193 Bellaterra, Spain

Correction to: *Scientific Reports* 10.1038/s41598-018-24392-1, published online 12 April 2018

In the Supplementary Information file originally published with this Article, an equation was omitted. This error has been corrected in the Supplementary Information that now accompanies the Article.

