# Supplementary material for: Angular flux creep contributions in YBa2Cu3O7−δ nanocomposites from electrical transport measurements
Source: Sci Rep. 2018 Apr 12;8:5924. doi: 10.1038/s41598-018-24392-1 (PMC5897461; doi:10.1038/s41598-018-24392-1)
Supplement: Supplementary file 1 — Supplementary Information [file 41598_2018_24392_MOESM1_ESM.pdf]

# Supplementary Information for: Angular flux creep contributions in $YBa_2Cu_3O_{7-\delta}$ nanocomposites from electrical transport measurements

***F. Vallès, A. Palau, V. Rouco, B. Mundet, X. Obradors, T. Puig***

***Institut de Ciència de Materials de Barcelona, ICMAB-CSIC, Campus UAB, 08193 Bellaterra, Spain***

The normalized magnetic relaxation rate S was calculated by inductive methods with a superconducting quantum interference device (SQUID) magnetometer from the logarithmic decay of $J_c$ with time: S= -d[ln($J_c$)]/d[ln(t)]. It was also calculated by electrical transport measurements from the equation S=1/(N-1), where N is the index of the power law relation $E \propto J^N$, calculated by a linear fit of the log-log E-J plot where log(E)=N*log(J)+C. The standard error of the slope δN is propagated to the standard error of the creep rate δS, following the equation:

$$\delta S = \frac{1}{(N-1)^2}\delta N$$

From the comparison of both methods depicted in Fig. S1, it is worth mentioning that similar features are observed. However, at low temperatures S values from electrical transport are slightly higher, whereas at high temperatures, the plastic-creep regime, where the S parameter increases abruptly, is observed at lower temperatures with SQUID magnetometry. This discrepancy stems from the use of different criteria and the distinctive vortex dynamics involved in each experiment.

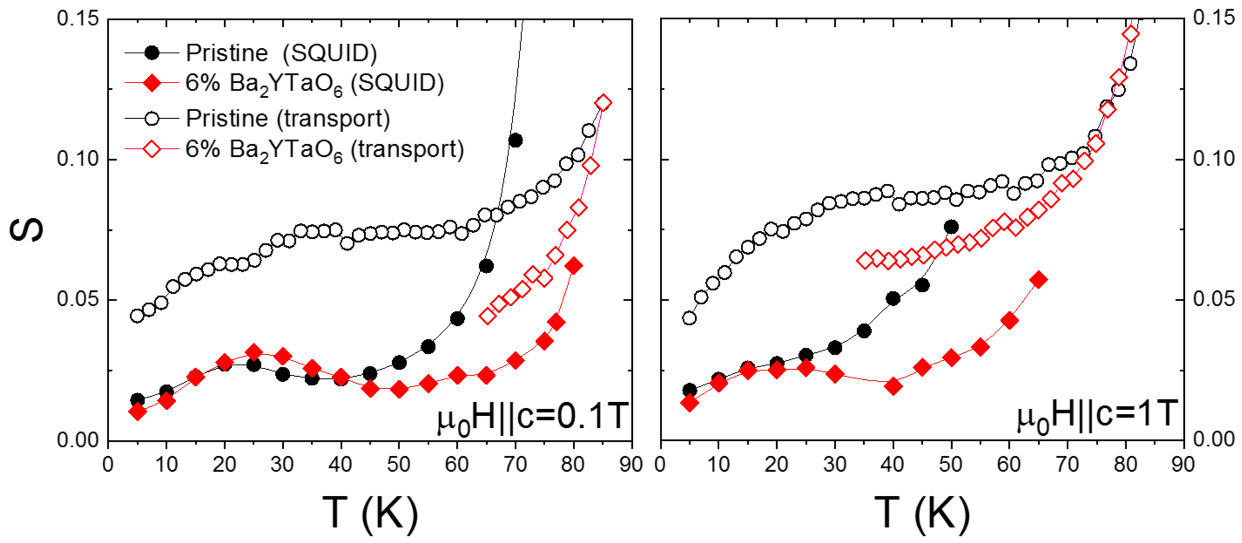


**Figure S1. Temperature dependence of S from electric transport and SQUID magnetometry for H||c.** The curves were measured for two pristine samples (circles) and two nanocomposites (diamonds) at 0.1 T and 1 T by electrical transport measurements (open) and by SQUID magnetometry (closed).

Fig. S2 shows the Scanning Transmission Electron Microscope (STEM) images for a pristine, nanocomposite C and nanocomposite D, presenting stacking fault densities of $3.2{\cdot}10^{-4}$ $nm^{-2}$, $6.4{\cdot}10^{-4}$ $nm^{-2}$ and $2.2{\cdot}10^{-3}$ $nm^{-2}$ respectively. These densities have been calculated by dividing the number of identified stacking faults by the total YBCO area, as shown in Fig. S2 (d).

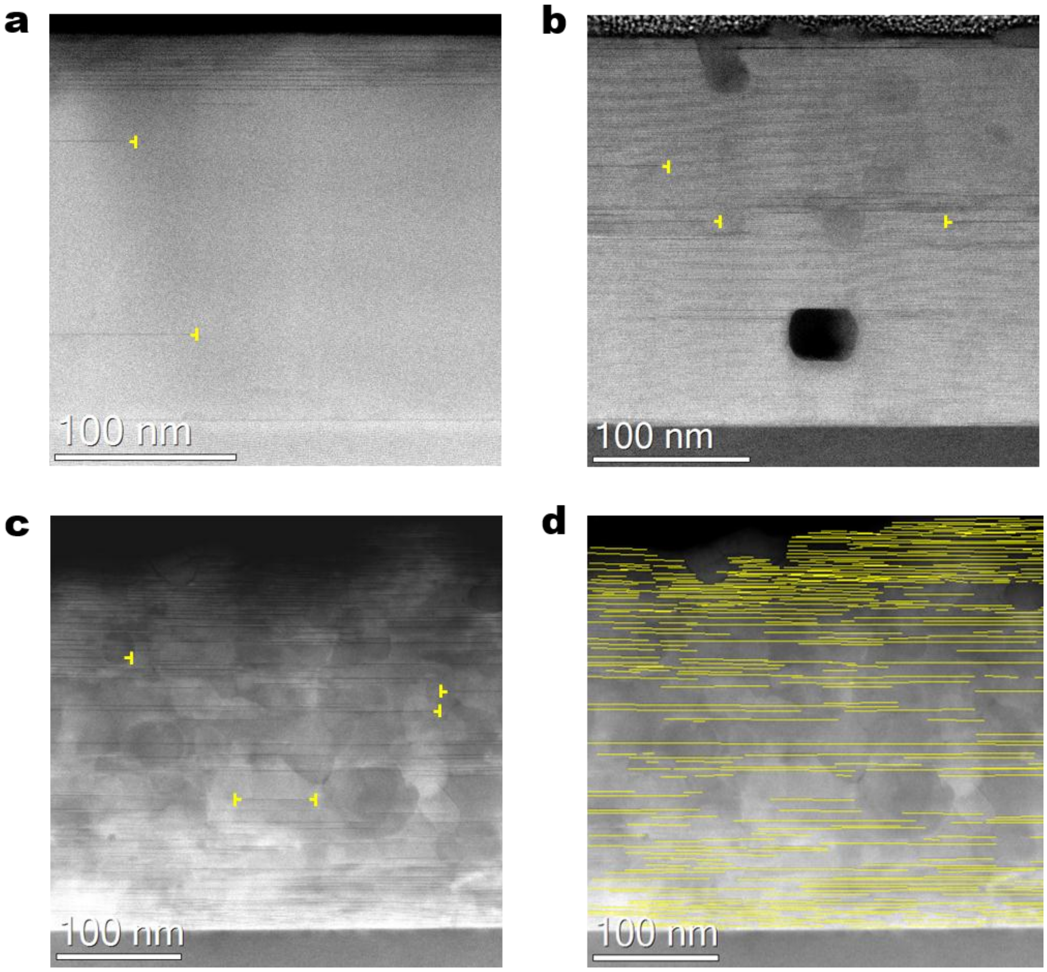


**Figure S2. STEM characterization of films with different stacking fault densities.** Z-contrast low magnification images for **(a)** a pristine film, **(b)** nanocomposite C and **(c)** nanocomposite D, showing 248-intergrowths as black stripes. Yellow symbols point out the edges of some of the 248-intergrowths in each film. The identification of the total amount of intergrowths of nanocomposite D is marked in yellow stripes in image **(d)**.

Different ranges of electric field close to the critical electric field $E_c$ have been tested for a pristine film in Fig. S3 (a) in order to find the range with less standard error $\delta S$ and to evaluate the variation of S. A minimum electric field $E_{min}$ of 50 $\mu$V/cm provides the fitting range with less standard error (Fig. S3 (b)), whereas the maximum discrepancy of S between different fitting ranges is found to be ~10% at low fields (Fig. S3 (c)).

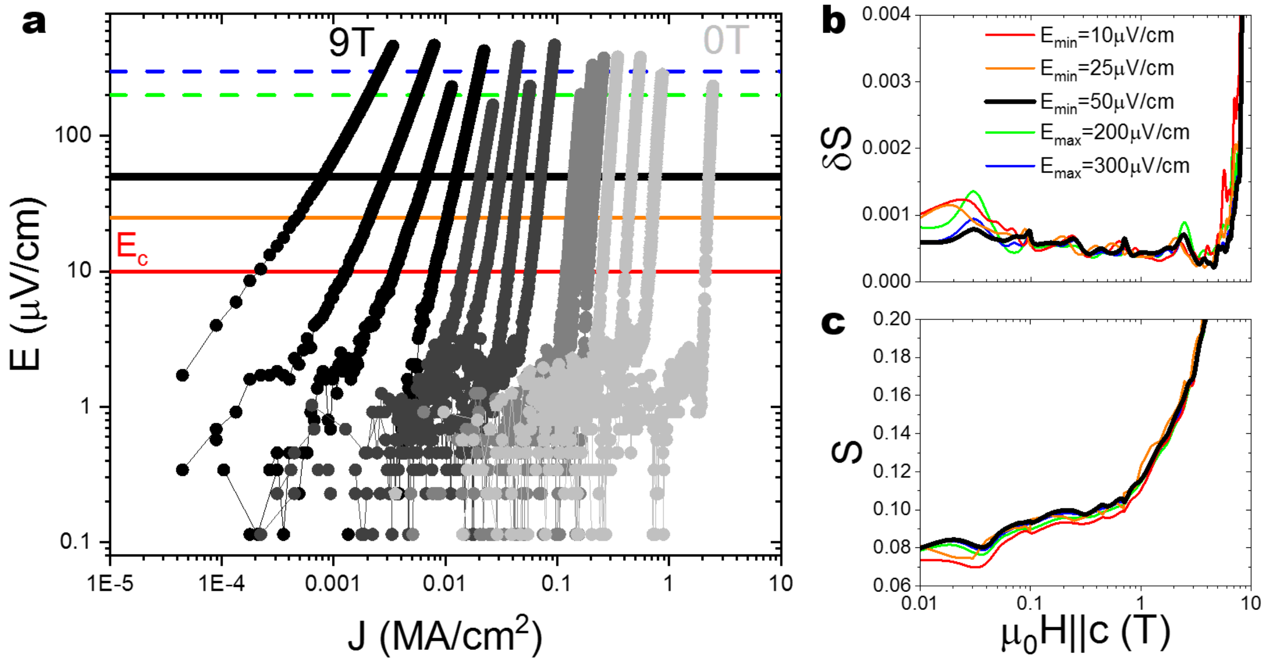


**Figure S3. Linear fit of E-J characteristics.** On the left, **(a)** electric field-current density characteristics for the pristine sample from Fig. 1 at 77 K, at 0 T, 0.1 T, 0.2 T, 0.4 T, 0.6 T, 0.8 T and from 1 T until 9 T for H||c. The solid lines represent the $E_{min}$ used for the fitting range beginning at $E_{min}$ and ending at the highest point of each curve. The dashed lines represent the maximum electrical field $E_{max}$ used for a fitting range beginning at $E_{min}$=50$\mu$V/cm and ending at $E_{max}$. On the right, magnetic field dependence at 77K of **(b)** $\delta S$ and **(c)** S obtained by applying the different fitting ranges.
